# Impact of BMI *z*-score on left ventricular mechanics in adolescent girls

**DOI:** 10.3389/fped.2023.1165851

**Published:** 2023-07-26

**Authors:** Justine Paysal, Etienne Merlin, Emmanuelle Rochette, Daniel Terral, Stéphane Nottin

**Affiliations:** ^1^ LaPEC UPR 4278, Laboratory of Cardiovascular Physiology, Avignon University, Avignon, France; ^2^ Néonatologie et Réanimation Pédiatrique, CHU Clermont-Ferrand, Clermont-Ferrand, France; ^3^ CHU Clermont-Ferrand, Department of Pediatrics, Clermont-Ferrand, France; ^4^ INSERM, CIC 1405, Unité CRECHE, Université Clermont Auvergne, Clermont-Ferrand, France

**Keywords:** BMI *z*-score, anorexia nervosa, obesity, myocardial strains, speckle-tracking echocardiography, adolescent

## Abstract

**Background:**

Adolescent weight disorders ranging from anorexia nervosa (AN) to obesity (OB) can impact the heart by causing opposite alterations in its morphology, suggesting a direct impact of body mass index (BMI) on the heart. Cardiac function is relatively preserved as assessed by standard echocardiography. However, few studies have used 2D speckle-tracking echocardiography (2D-STE), which can detect subtle alterations of left ventricular (LV) function by evaluating deformations. This study aimed to assess the link between the BMI *z*-score of adolescent girls and myocardial function.

**Methods:**

Ninety-one adolescent girls comprising 26 AN patients (age 14.6 ± 1.9 years), 28 OB patients (age 13.2 ± 1.4 years), and 37 controls (age 14.0 ± 2.0 years) underwent 2D-STE to assess LV morphology and myocardial global and regional deformations.

**Results:**

The BMI *z-*score of our population ranged from −4.6 to 5.2. LV morphological remodeling was significantly and positively correlated with the BMI *z*-score (*R*^2^ = 0.456, *p* < 0.0001 for LV mass). Global longitudinal strain (LS) and regional LS recorded at the mid and apical levels were significantly correlated with the BMI *z*-score (*R*^2^ = 0.196, *p* = 0.0001 and *R*^2^ = 0.274, *p* < 0.0001, respectively, for apical and medial LS). Circumferential strains and twisting mechanics were not correlated with the BMI *z*-score. Fibrinogen and systolic blood pressure were the main variables explaining the alteration of LS.

**Conclusion:**

We observed that the BMI *z*-score had an impact on LV mechanics, especially on medial and apical LS. Neither circumferential nor twisting mechanics were altered by the BMI *z*-score in adolescent girls.

## Introduction

Weight disorders (WDs), ranging from anorexia nervosa (AN) to overweight (OW) and obesity (OB), are extremely common in adolescence (1, 2). AN, defined by a marked weight loss and low body mass index (BMI), affects 3% of European women and occurs most often in girls aged 12–25 years (1). OB, characterized by excess weight and high BMI, is very common in the pediatric population (17% in the United States) (3), with a high prevalence among those aged 15–19 years (2). These pathologies have numerous impacts on the cardiovascular system, illustrated, for example, by a decrease in heart rate (HR) and blood pressure (BP) in AN (4) and, conversely, an increase in OB (2, 5). Cardiac remodeling is associated with a decrease in the left ventricular mass (LVM) and LV wall thickness in AN (4, 6) and, conversely, an increase in LVM and LV wall thickness in OB (2, 5, 7). In both cases, structural alterations were observed, with the presence of myocardial fibrosis (1, 8, 9) probably linked in part to chronic low-grade systemic inflammation (9, 10). Despite these structural and morphological changes, global cardiac function, assessed by standard ultrasound methods, appears relatively preserved (2, 4, 5, 7). Several recent studies (5, 7, 11–13) assessed regional myocardial strains and twisting mechanics (this latter being an important determinant of LV systolic and diastolic function) (14) by speckle-tracking echocardiography (STE) to characterize their LV function with greater sensitivity than conventional echocardiography (15). The first findings suggested a decrease in LV longitudinal strains in OB (5, 7, 12, 13, 16) potentially counterbalanced by an increase in twisting mechanics (16), underlining possible links between WD and cardiac mechanics.

Importantly, the above studies provided minimal data on AN patients (11) or focused on OB patients only (5, 10, 13). To our knowledge, no study has evaluated the impact of WD on cardiac morphology and regional myocardial function over a broad scale ranging from low BMI in AN to high BMI in OB patients. Interestingly, cardiac mechanics might be altered on a continuum, owing to the opposite changes in loading conditions (i.e., BP) and cardiac morphological remodeling (i.e., cardiac hypotrophy vs. hypertrophy) reported in adolescents with WD, but this remained to be confirmed.

We set out to evaluate the cardiac repercussions of WD over a wide range of BMI in adolescents and to explore the links between LV variables (including regional LV strains and twisting mechanics) and BMI z-score. The latter cancels out the changes in BMI that occur with advancing age in adolescents (17). Since AN affects women preferentially (18), we focused on a population of women. We hypothesized that in adolescent girls with WD, (i) there would be a continuum in the adaptation of LV morphology and function, with strong correlations between LV size, myocardial strains, twist, and BMI *z*-score, and (ii) these modifications of strains would be secondary to changes in load conditions, cardiac morphological adaptations, and a particular inflammatory profile.

## Methods

### Study population

This prospective study comprised adolescent girls with AN, normal weight, OW, or OB aged 10–18 years to obtain a broad continuous scale of BMI *z*-scores. The patients were diagnosed in a pediatric department of a university hospital in France between March 2019 and January 2020. AN patients fulfilled the Diagnostic and Statistical Manual (DSM) V criteria for AN (American Psychiatric Association) (19). OB patients met the International Obesity Task Force (IOTF) C30 criteria (20). The adolescents with OB included had primary obesity (i.e., not secondary to any genetic or endocrine pathology) and did not present diabetes or dyslipidemia (verified on the same day as echocardiography by a blood test). The BMI *z*-score was calculated for all participants. None had congenital heart defects, arrhythmia, myopathy, rheumatic pathology, or positive family history of cardiac disease. Written informed consent was obtained from the study participants and their guardians. The Ile-de-France Ethics Committee approved the protocol for this study (18.12.05.66738 CAT 2). Moreover, all methods were carried out in accordance with relevant guidelines and regulations.

### Anthropometric and clinical assessments

Body height and body mass were measured. BMI was calculated as body mass/body height^2^. Body surface area (BSA) was calculated according to Boyd (21). Systolic and diastolic blood pressures (SBP and DBP, respectively) were measured using an automatic device (General Electric, Dynamap PRO 300 V2, Boston). The average resting HR was recorded at night using a validated HR monitor (Polar V800, Polar, Finland) (22) and a chest strap (Polar H10, Polar, Finland).

### Echocardiographic recordings

Echocardiography was carried out with the subject lying in the left lateral decubitus position by Vivid ultrasound systems (GE Healthcare, Horten, Norway) using a 3.5-MHz transducer (M4S probe). Cine loops were recorded in parasternal long-axis and apical (5, 4, 3, and 2 chamber) views and saved for blinded offline analysis (EchoPac, BT203 version, GE Healthcare). Grayscale images were saved at a frame rate of 80–90 frames/s, and color tissue velocity images were saved at a frame rate of 120–140 frames/s. 2D echocardiographic measurements were performed in accordance with the guidelines of the American Society of Echocardiography (23). All echocardiographic data were averaged from measurements obtained on 3–5 cardiac cycles.

### Cardiac morphology

LV diameters and myocardial thicknesses in diastole were measured from the parasternal long-axis view. LV mass was estimated using the Devereux formula and indexed for height^2.7^ as recommended for the pediatric population (24). LV volumes were assessed using Simpson's biplane method. Epicardial fat thickness (EFT) was measured according to recommendations suggested by Iacobellis (25). EFT was identified as the relatively echo-free space between the outer wall of the myocardium and the visceral layer of pericardium and measured perpendicularly on the free wall of the right ventricle at end-systole (25).

### LV systolic and diastolic functions

LV diastolic function was assessed from peak early (E wave) and atrial (A wave) transmitral flow velocities. Peak *e*′_lat_ recorded on the lateral wall was considered as an index of LV relaxation. *E*/*e*′_lat_ was used as an index of LV filling pressure (26). Ejection fraction (EF) was assessed using Simpson's biplane method. GLS, circumferential (CS), and twisting mechanics (apical and basal rotations, peak twist and untwisting rates) were obtained as previously detailed (27). Briefly, after tracing the endocardial border on the end-systolic frame of the 2D sequence, the software automatically tracked myocardial motion. Whenever the software signaled poor tracking efficiency, the observer adjusted the endocardial trace line, the regional width of the region of interest, or both until a better tracking score could be obtained. Then, EchoPAC data were exported as “.txt files” to be processed by a specific toolbox (Scilab version 4.1; Consortium Scilab, INRIA-ENPC, Paris, France). To adjust all strain parameters for intersubject differences in heart and frame rates, the time sequence was also normalized to the percentage of systolic duration (i.e., time was 100% at end-systole). LV twist was calculated as the difference between apical and basal rotations at each percentage of systolic duration. We considered GLS, CS, and LV rotations, and peak twist as indexes of myocardial systolic function. Regional analysis was carried out at the level of the longitudinal strains (basal, median, apical LS) and circumferential (basal and apical CS).

### Biological data

A fasting venous blood sample was taken for biochemical determinations by an automated immunoassay of fibrinogen.

### Statistical analyses

Statistical analyses were performed using SPSS 25 statistical software. All values were expressed as mean ± SD. Before the study, we calculated the size of the groups to differentiate the GLS with a statistical power of 90% and an effect size of 1, which correspond to a difference of the GLS of 2% with an SD of 2% and taking into account the Bonferroni correction when including three groups in the analyses. The analysis indicated a minimal size group of 26 patients in each group. One-way analysis of variance (ANOVA) was used to compare groups after checking the normality of distribution of each variable by a Shapiro–Wilk test. The non-parametric Kruskal–Wallis test was used when the variables do not meet the criteria of normal distribution. Correlations were determined between BMI *z*-scores and hemodynamic, biological, and cardiac ultrasound variables by linear regression analysis and Pearson’s correlation coefficient. *R*^2^ was calculated to assess the proportion of variance explained. Stepwise regressions, including hemodynamic, cardiac morphology, and biological variables, were then carried out. Statistical significance for all analyses was assumed at *p* < 0.05.

## Results

### Population characteristics and resting echocardiography

Ninety-one adolescent girls with BMI *z*-scores ranging from −4·6 to 5·2 comprising 26 AN, 33 normal-weighted, 4 OW, and 28 OB patients were included. Table 1 shows the anthropometric characteristics and the standard echocardiographic variables of our populations. LV wall thicknesses and mass were higher in OB. EF was similar between groups. The diastolic function of AN patients was characterized by a lower A wave, whereas that of OB patients was characterized by a higher E wave and a higher *E*/*e*′_lat_ ratio. Table 2 shows the 2D speckle tracking echocardiography parameters of our populations. GLS was higher in AN patients compared to that in controls and adolescents with obesity. Their median LS was higher compared to controls, while apical and basal LSs were similar. In obese adolescents, median and apical LSs were lower, while basal LS was similar to that in controls. There was no difference in the circumferential strain, rotations, and twists between groups.

**Table 1:** General characteristics and standard ultrasound parameters of AN patients (*n* = 26), controls (*n* = 37), and obese adolescents (*n* = 28).

	Anorexics (*n *= 26)	Controls (*n *= 37)	Obeses (*n *= 28)
Age (years)	14.6 ± 1.9	14.0 ± 2.0	13.2 ± 1.4^#^
Anthropometry			
Height (cm)	159.8 ± 9.1	162.7 ± 9.6	162.9 ± 5.7
Body mass (kg)	40.7 ± 8.2***	53.3 ± 11.4	90.4 ± 13***^,###^
BMI (kg m^−2^)	15.84 ± 2.06***	19.98 ± 3.17	34.02 ± 4.10***^,###^
BMI *z* score	−1.8 ± 1.1***	0.4 ± 1.3	4.1 ± 0.6***^,###^
BSA (m^2^)	1.33 ± 0.16***	1.55 ± 0.20	2.08 ± 0.17***^,###^
Echocardiography parameters			
LV septum thickness (cm)	0.72 ± 0.15	0.76 ± 0.11	0.83 ± 0.18^#^
LV posterior wall thickness (cm)	0.67 ± 0.12***	0.74 ± 0.11	0.88 ± 0.13***^,###^
LV mass (g)	79 ± 26***	96 ± 24	128 ± 29***^,###^
LV mass^2.7^ (g m^2.7^)	22 ± 6***	26 ± 5	34 ± 7***^,###^
LV end-diastolic volume (ml)	79 ± 19	90 ± 23	119 ± 27***^,###^
LV end-systolic volume (ml)	29 ± 8	33 ± 9	42 ± 11***^,###^
Ejection fraction (%)	64 ± 5	64 ± 6	64 ± 6
E wave (cm s^−1^)	84 ± 17	82 ± 14	90 ± 11*
A wave (cm s^−1^)	30 ± 6***	40 ± 8	39 ± 7^###^
*E*/*A*	2.9 ± 0.9***	2.1 ± 0.5	2.4 ± 0.5***^,###^
*e*′_lat_ (cm.s^−1^)	13.0 ± 1.4	13.5 ± 1.6	13.2 ± 1.6
*E*/*e*′_lat_	5.1 ± 1.0	4.7 ± 0.8	5.2 ± 0.9*

BMI, body mass index; BSA, body surface area; LV, left ventricular. Values are mean ± SD.

* Significantly different from controls *p* < 0.05.

*** Significantly different from controls *p* < 0.001.

^#^ Significantly different from anorexics *p* < 0.05.

^###^ Significantly different from anorexics *p* < 0.001.

**Table 2:** 2D speckle tracking echocardiography parameters of AN patients (*n* = 26), controls (*n* = 37), and obese adolescents (*n* = 28).

	AN patients (*n *= 26)	Controls (*n *= 37)	Obeses (*n *= 28)
Longitudinal strains			
GLS (%)	−18.8 ± 2.0**	−16.9 ± 2.8	−16.8 ± 1.9^##^
Basal LS (%)	−17.9 ± 2.6	−16.9 ± 2.2	−17.8 ± 2.1
Median LS (%)	−21.3 ± 2.0***	−20.1 ± 2.0	−18.2 ± 2.1***^,###^
Apical LS (%)	−20.1 ± 3.4	−18.4 ± 3.2	−15.8 ± 3.5***^,###^
Circumferential strains			
GCS (%)	−25.8 ± 2.9	−27.0 ± 3.4	−25.5 ± 4.0
Basal CS (%)	−21.9 ± 2.9	−23.1 ± 3.6	−21.9 ± 4.2
Apical CS (%)	−29.6 ± 4.6	−30.9 ± 4.4	−29.1 ± 6.5
Rotations			
Basal rotation (°)	−3.3 ± 1.7	−3.7 ± 2.0	−4.0 ± 2.5
Apical rotation (°)	5.8 ± 2.6	5.7 ± 2.1	5.5 ± 2.9
Twist (°)	7.3 ± 3.2	7.5 ± 3.3	8.3 ± 4.1

GLS, global longitudinal strain; GCS, global circumferential strain. Values are mean ± SD.

** Significantly different from controls *p* < 0.01.

*** Significantly different from controls *p* < 0.001.

^##^ Significantly different from anorexics *p* < 0.01

^###^ Significantly different from anorexics *p* < 0.001.

### Correlations between BP, HR, fibrinogen, and BMI *z*-score

**Figure 1:**
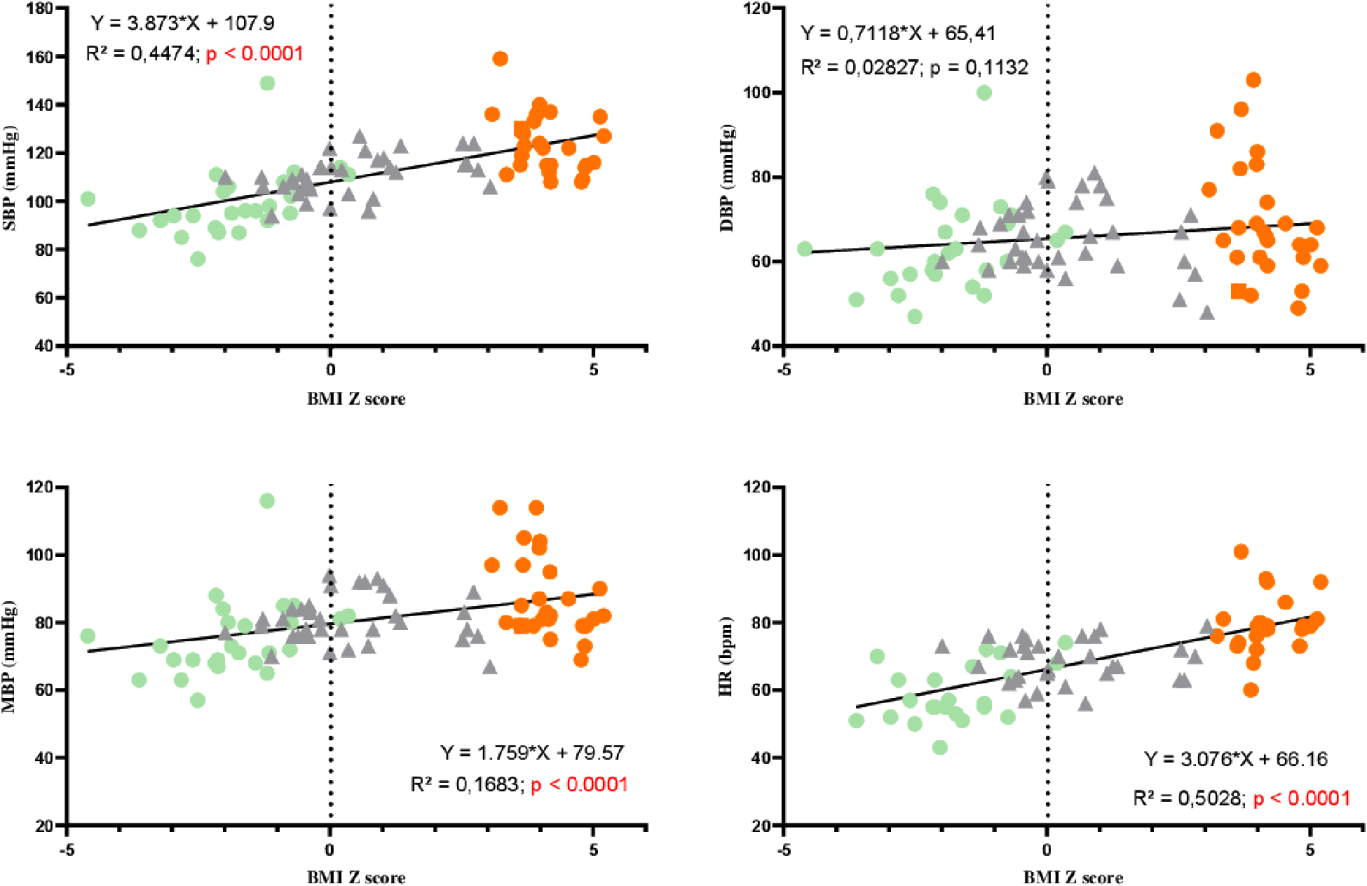
Correlations between blood pressure, heart rate, and BMI *z*-score in AN patients (green circles), controls (gray triangles), and adolescents with obesity (orange circles). SBP, systolic blood pressure; DBP, diastolic blood pressure; MBP, mean blood pressure; HR, heart rate.

**Figure 2:**
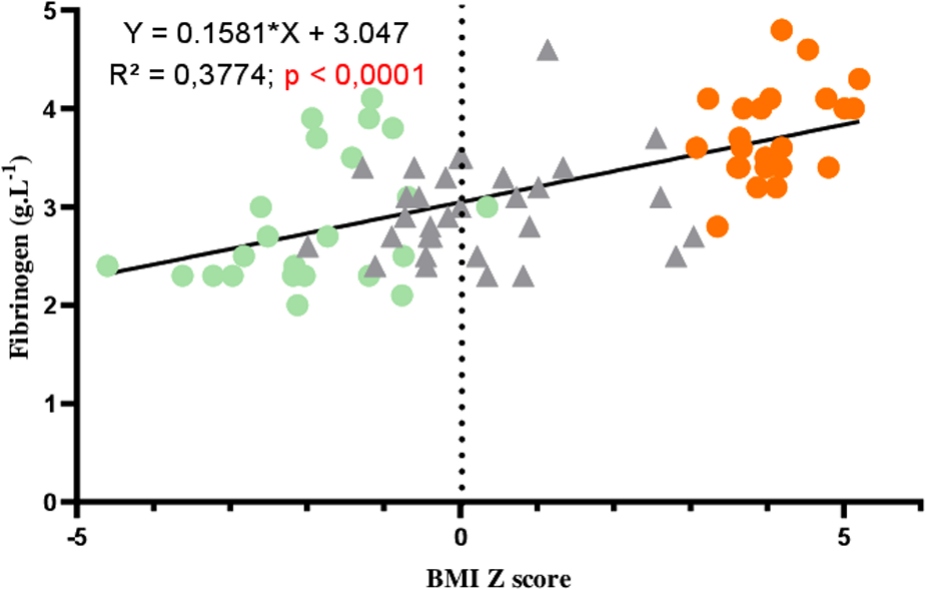
Correlation between fibrinogen and BMI *z*-score in AN patients (green circles), controls (gray triangles), and adolescents with obesity (orange circles).

Figure 1 shows the correlations between BP, HR, and BMI *z*-score. Significant correlations were observed between systolic BP, HR, and BMI *z*-score but not with diastolic BP. Figure 2 represents the positive correlation between fibrinogen and the BMI *z*-score.

### Correlations between LV morphological and function variables and BMI *z*-score

**Figure 3:**
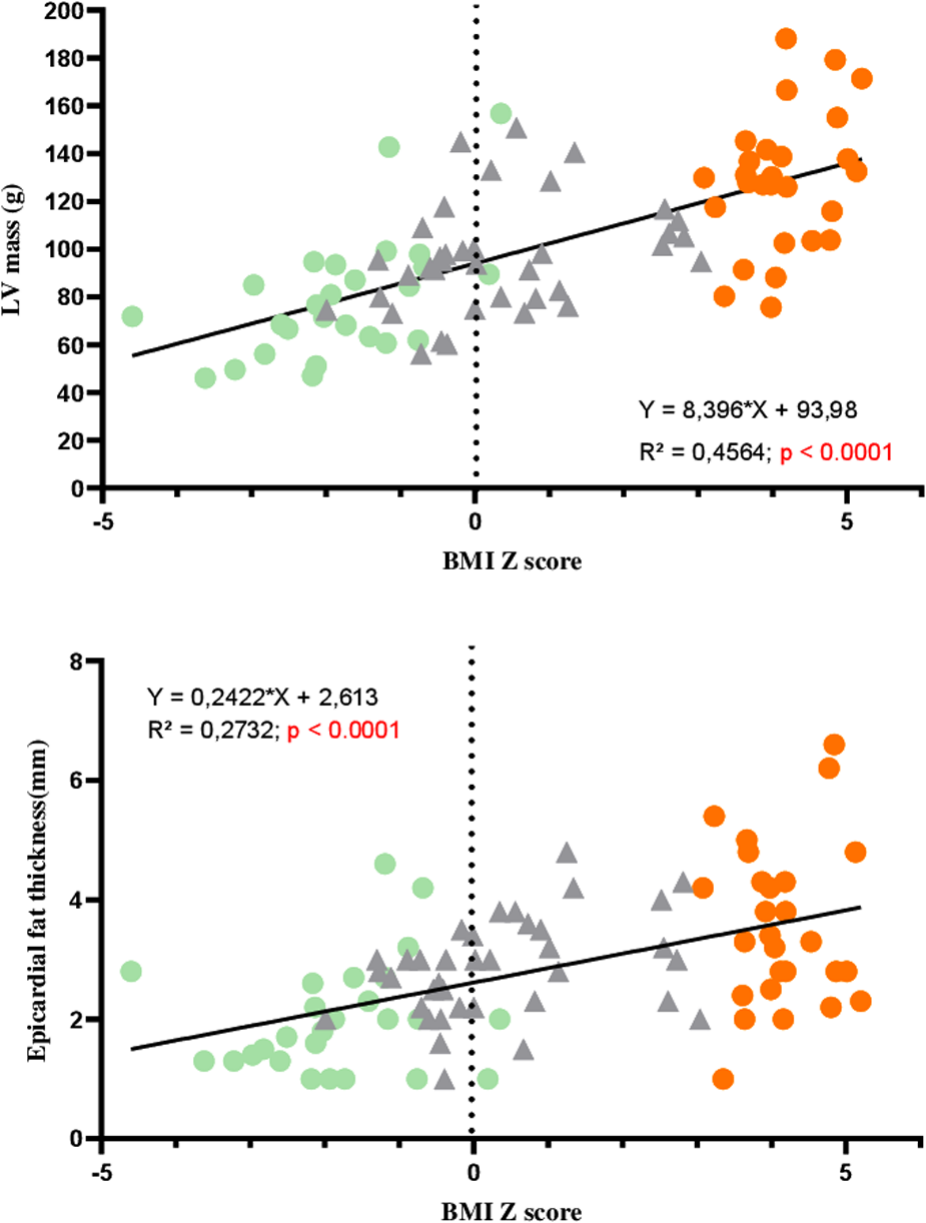
Correlations between LV mass, epicardial fat thickness, and BMI *z*-score in AN patients (green circles), controls (gray triangles), and adolescents with obesity (orange circles).

**Figure 4:**
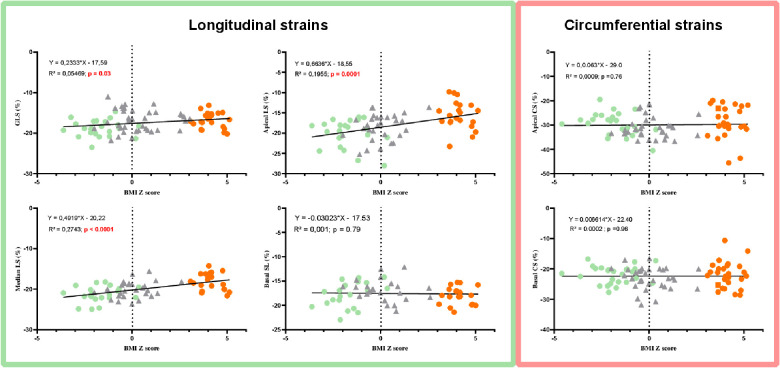
Correlations between LV longitudinal and circumferential strains and BMI *z*-score in AN patients (green circles), controls (gray triangles), and adolescents with obesity (orange circles).

**Figure 5:**
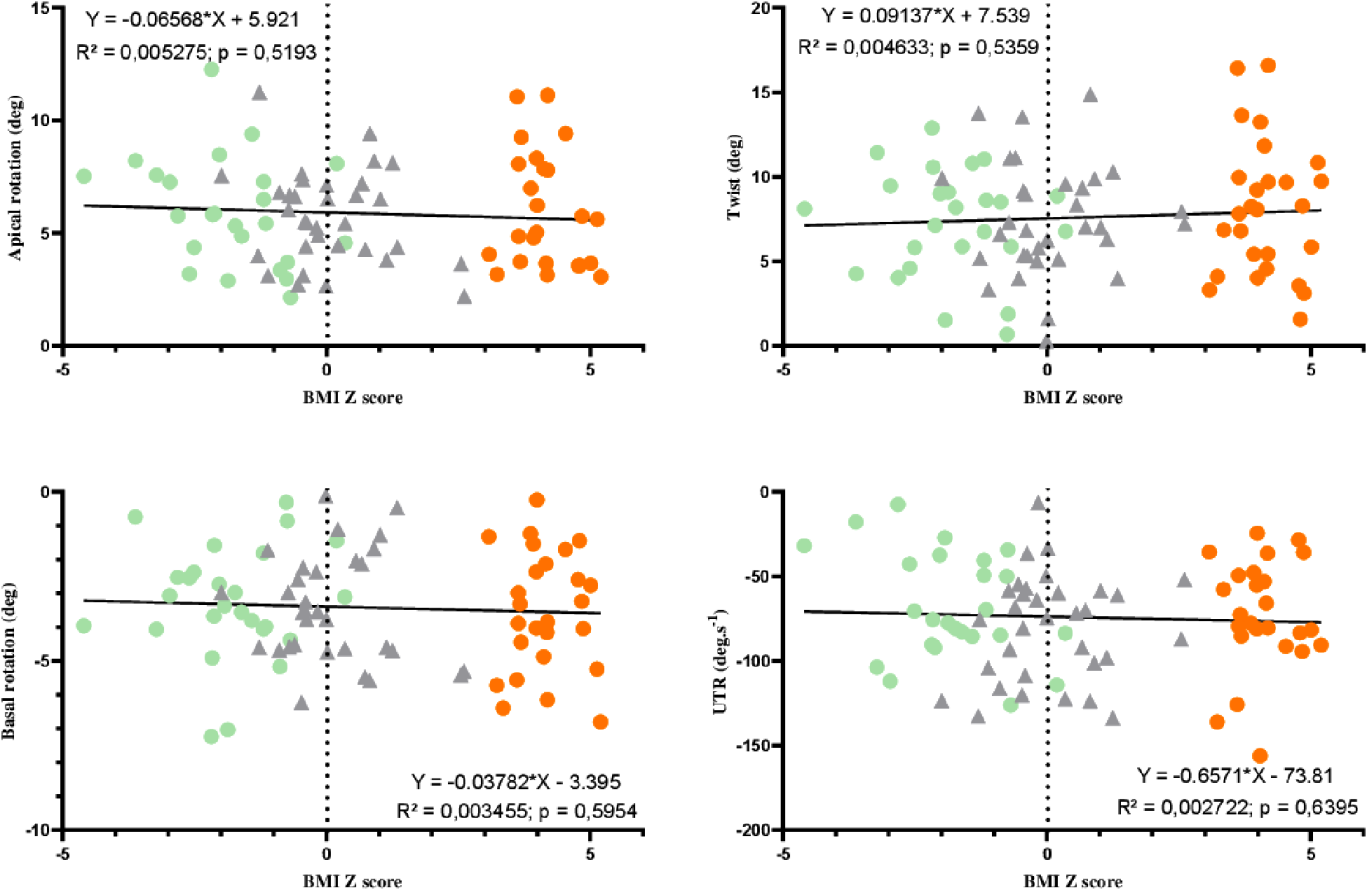
Correlations between twisting mechanics parameters and BMI *z*-score in AN patients (green circles), controls (gray triangles), and adolescents with obesity (orange circles).

LV mass and EFT were significantly correlated with the BMI *z*-score (Figure 3). Significant correlations were found between GLS and the BMI *z*-score, with the highest GLS being observed for the lowest BMI *z*-score. Interestingly, correlations were obtained between BMI *z-*scores and longitudinal strains at the apical and medial levels but not at the base. No significant correlations were observed between BMI *z*-scores and circumferential strains (Figure 4). Similarly, no significant correlations were observed between the LV basal and apical rotations, twist and untwisting rates, and the BMI *z*-scores (Figure 5).

### Univariate and stepwise regression analyses

In univariate regression analyses, SBP, HR, LV mass, EFT, fibrinogen, and NT-proBNP were significantly correlated with median LS. EFT, fibrinogen, and NT-proBNP were significantly correlated with apical LS.

In stepwise regression analyses, fibrinogen and SBP could explain the changes in median LS, and fibrinogen alone could explain the changes in apical LS.

## Discussion

Our study aimed to assess the link between the BMI *z*-score of adolescent girls with WD and myocardial function. Our main results were that (i) LV morphological remodeling was significantly and positively correlated with the BMI *z*-score, (ii) GLS and longitudinal strains recorded at the apical and medial levels but not at the base were significantly correlated with the BMI *z*-score, with the highest strains being observed in adolescents with the lowest BMI *z*-scores, (iii) from short-axis planes, both circumferential strains and rotations, and so twist and untwisting rates, were not correlated with the BMI *z*-score, and (iv) from a multivariate stepwise regression analysis, fibrinogen and SBP were the main variables explaining the alteration of regional longitudinal strains with the BMI *z-*score.

### Cardiac remodeling and BMI *z*-score

We found a positive correlation between the BMI *z*-score and LV mass. These results are in agreement with current knowledge on the cardiac remodeling induced by WD, namely, a decrease in measurements in AN (4, 28, 29) and, conversely, an increase in OB (2, 7). These morphological adaptations are linked not only to the nutritional state but also to the load conditions (30), which vary with BMI, as demonstrated in our study by a positive correlation between the BMI *z-*score and clinical variables of HR and BP. Therefore, in AN, possible causes of hypotrophy include decreased afterload from relative hypotension, leading to downregulation of the LV mass (30), reduced preload, leading to ventricular remodeling, and a direct effect of malnutrition, similar to that observed in skeletal musculature (30). By contrast, in OB, the excess adipose tissue is responsible for insulin resistance with deleterious effects on the heart: insulin may aggravate hypervolemia through salt and water retention, act as a myocardial growth factor, activate the sympathetic nervous system, and therefore ultimately increase the size of the heart (7). On the other hand, the increase in preload and afterload (linked to arterial stiffness) also seems to be involved in the morphological changes (2). Moreover, in line with the literature (25), we showed that these morphological changes were associated with the thickening of epicardial fat, which is closely correlated with the BMI *z*-score. Interestingly, this epicardial fat is considered a marker of cardiovascular risks (25), in particular, because, in excess, it has direct effects on coronary atherosclerosis and causes fatty infiltration, inflammation, and myocardial fibrosis (9, 25).

### Regional myocardial function and BMI *z*-score

The EF, assessed from conventional echocardiographic variables, remained unchanged, whatever the BMI *z*-score. The strength of our study was to evaluate systolic function from the GLS measured by STE. Interestingly, GLS was negatively correlated with the BMI *z*-score (i.e., lower strains with higher BMI *z*-scores). Moreover, the regional assessment of LV longitudinal strains gave additional information by demonstrating that these correlations were observed only at the mid and apical levels but not at the base. Our results thus robustly demonstrate a continuum of adaptation of longitudinal strains at mid and apical levels in a population of adolescents from very low to high BMI *z*-scores. In AN patients, only one study used STE and found no alteration in GLS compared with controls, except in a very small subgroup of patients with purging behavior in whom apical longitudinal strains were reduced. They demonstrated that the only predictor of the apical strains was the BMI *z*-score (11). In adolescents with obesity, it has been well established that GLS is lower than that in controls (5, 7, 12, 13, 31) and that the latter is negatively correlated with the BMI *z*-score (31). Of note, from regional analysis of longitudinal strains, Binnetoglu et al. (12) also reported a more pronounced decrease in strains at the mid and apical segments compared to the basal level. To gain insight into the underlying mechanisms of these alterations, we performed multivariate stepwise regression analyses and observed that fibrinogen and SBP were the two main independent predictors of LV mid or apical strains. Fibrinogen was considered a high-risk marker for developing vascular inflammatory diseases, such as arterial hypertension and atherosclerosis (32). Our results demonstrated close correlations between fibrinogen and the BMI *z*-score and the SBP. A chronic inflammatory state may therefore appear with increasing BMI *z*-score mediated, in particular, by fibrinogen, which promotes the appearance of higher SBP, afterload, and *a fine* reduction in strains. It has been recently demonstrated that longitudinal strains are heavily influenced by afterload (33). The inflammatory state could also induce an adverse effect on strains via the occurrence of myocardial fibrosis, which is well established in OB (9).

### Twisting mechanics and BMI *z*-score

For a comprehensive evaluation of the impact of BMI *z*-score on the myocardial mechanics, we also assessed circumferential strains and rotations. Rakha et al. previously observed a decrease in global circumferential strains in obese children compared to that in controls (31). Interestingly, in our study, both circumferential strains and rotations (and so twists) (14) were normal regardless of the BMI *z*-score. The discrepancies between findings obtained on longitudinal and circumferential strains suggested a specific impact of BMI *z*-score on the longitudinal deformations. The greater vulnerability of longitudinally orientated myocardial fibers may be explained by their major distribution in the subendocardium, making them more susceptible to wall stress, ischemia, and fibrosis (34).

The LV twist helps to create a uniform distribution of LV fiber stress and fiber shortening across the wall and is essential for the course of systole. Its disappearance has been shown to increase oxygen demand and reduce the efficiency of LV systolic function (14). The LV twist is affected by loading conditions of the heart; an increase in afterload and/or a decrease in preload induce a decrease in twist in animal studies (14). In physiological states in which pre- and afterload were decreased, the twist remained unchanged, or was increased (14). In our study, the wide range of BMI *z-*scores, accompanied by changes in SBP, did not impact the twist. We note that other factors could influence the twist and allow its conservation, such as an increase in intrinsic myocardial contractility or an increase in sympathetic activity, which have been suggested to increase the twist (14, 27).

The energy stored during the LV twist in elastic components is restored very early in diastole, creating an intraventricular pressure gradient that favors LV filling, thus linking systole to diastole (systolic–diastolic coupling) (14). Here, we observed no significant correlation between the peak untwisting rate and the BMI *z*-score, suggesting that this mechanism was preserved over a wide range of WD. Taken together, our results strongly support that twisting mechanics are unaffected by variations in the BMI *z*-score. The maintenance of twisting mechanics could probably explain in part the preservation of systolic and diastolic function observed from conventional echocardiography in adolescents with WD, regardless of their BMI *z*-scores.

### Study limitations

To obtain a broad, continuous scale of the BMI *z*-score, we decided to include AN patients who were not constitutionally thin. This could therefore introduce bias in our results linked to the pathophysiology specific to this disease. Moreover, echocardiography has inherent limitations of resolution that could affect speckle tracking, especially in the cohort of adolescents with obesity. However, the image quality obtained in the adolescents included in our study was quite good, and all 2D strain analyses were performed from good-quality cine-loop recordings.

## Conclusion

WDs ranging from AN to OB are particularly common in adolescents and result in a broad continuous scale of the BMI *z*-score. Our study showed a positive correlation between this anthropometric marker and the measurements of the LV. Moreover, GLS and apical and mid-longitudinal strains were negatively correlated with the BMI *z*-score. Conversely, basal longitudinal strain and other strains occurring during a cardiac cycle were not affected by the BMI *z*-score. Fibrinogen, and to a lesser extent SBP, could explain the modifications of the longitudinal strains. The effect of inflammatory status, mediated in part by fibrinogen, on LV function awaits confirmation in further studies.

## Data Availability

The raw data supporting the conclusions of this article will be made available by the authors without undue reservation.
